# Succinate Promotes M1 Polarization of Intestinal Macrophages in Mice With Necrotizing Enterocolitis Through the PI3K/AKT Pathway

**DOI:** 10.1002/pdi3.70026

**Published:** 2025-09-27

**Authors:** Sha Liu, Fang‐Ling Tang, Xiao‐Lin Yan, Xiao‐Chen Liu, Qing Ai, Lu‐Quan Li, Lei Bao

**Affiliations:** ^1^ Department of Neonatology National Clinical Research Center for Child Health and Disorders Ministry of Education Key Laboratory of Child Development and Disorders Key Laboratory of Child Rare Disease in Infection and Immunity Children's Hospital of Chongqing Medical University Chongqing China

**Keywords:** macrophage polarization, necrotizing enterocolitis, PI3K/AKT, succinate

## Abstract

Necrotizing enterocolitis (NEC) is a devastating gastrointestinal disorder that frequently affects premature infants, and its pathogenesis is closely related to macrophage polarization. This study investigated the effects of succinate, a metabolite of the intestinal flora, on macrophage polarization in NEC. Succinate aggravated intestinal injury caused by NEC and inhibited the proliferation of damaged mouse monocyte‒macrophage leukemia cells (RAW264.7 cells). It was confirmed by multiple methods that succinate intervention promotes the polarization of intestinal macrophages toward the M1 phenotype in neonatal NEC. This polarization was characterized by a significant upregulation of inducible nitric oxide synthase (iNOS) protein levels and *iNOS* mRNA expression, along with a marked suppression of arginase 1 (ARG1) protein levels and *Arg1* mRNA expression. Moreover, immunofluorescence analysis revealed that in the NEC intestine, the coexpression of the M1 macrophage marker F4/80^+^/CD86^+^ was significantly increased, whereas the coexpression of the M2 macrophage marker F4/80^+^/CD206^+^ was significantly decreased. Mechanistic studies revealed that succinate upregulated the expression levels of phosphorylated protein kinase B (*p*‐AKT) and hypoxia‐inducible factor 1 alpha (HIF1a) by activating the PI3K/AKT signaling pathway through its specific receptor succinate receptor 1 (SUCNR1). Further experiments revealed that the expression of polarization‐related markers in M1‐type macrophages was significantly suppressed after treatment with the SUCNR1‐neutralizing antibody or the PI3K inhibitor LY294002. These findings suggest that succinate may activate the phosphatidylinositol 3‐kinase/protein kinase B (PI3K/AKT) signaling pathway via SUCNR1 to promote the polarization of NEC macrophages toward the M1 phenotype, thereby accelerating NEC progression.

## Graphical abstract

Succinate promotes macrophage to the M1 phenotype in the mouse mononuclear macrophage leukemia cells (Raw264.7 cells) and neonatal mice with NEC. Succinate activates the phosphatidylinositol 3‐kinase/protein kinase B (PI3K/AKT) pathway via succinate recepter 1 (SUCNR1), promoting M1 polarization of NEC intestinal macrophages.

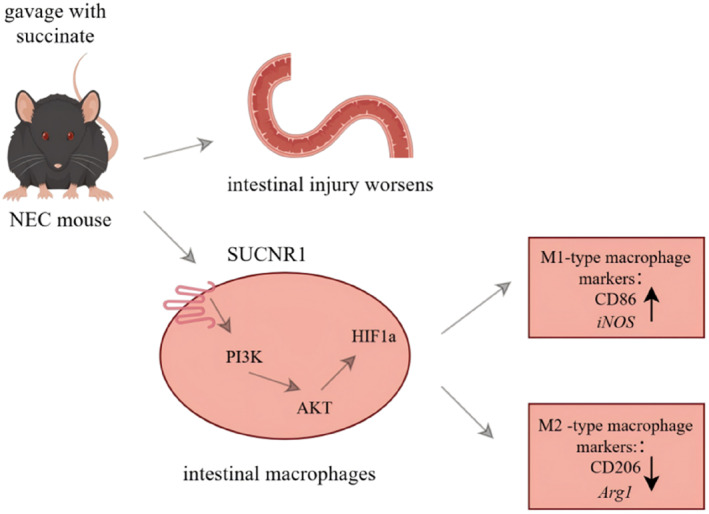

## 1 Introduction

Necrotizing enterocolitis (NEC) is a fatal gastrointestinal disease in the neonatal period characterized by vomiting, abdominal distention, and bloody stools [1, 2]. The prevalence is 5%–12% [3] and the prevalence is as high as 11.1% in very‐low‐birth‐weight infants [4]. Despite recent advances in medical technology, the mortality rate for NEC is still 23.5%, rising even higher in very‐low‐birth‐weight infants [5]. In addition, NEC can also lead to many long‐term complications, such as intestinal stricture, short bowel syndrome, growth retardation, intestinal failure, and neurodevelopmental disorders [6]. Exploring the new pathogenesis of NEC may provide potential new targets for the prevention and treatment of NEC.

Premature birth, abnormal microbial colonization, hypoxia/ischemia, and formula feeding [7, 8, 9] are recognized as high‐risk factors for NEC. In preterm infants, when attacked by the above high‐risk factors, the immune system overreacts, causing excessive inflammatory storms, leading to an impaired gut barrier, which can lead to NEC, in which macrophages play a crucial role [8, 10, 11]. Macrophages are important components of the innate immune system and constitute the first line of defense against pathogen invasion, and they can polarize to either the proinflammatory M1 or the anti‐inflammatory M2 phenotype depending on changes in the microenvironment [12]. Studies have shown that during the progression of NEC, intestinal macrophages polarize into the M1 phenotype, which is followed by the release of large amounts of cytokines that exacerbate the inflammatory response [13, 14, 15]; however, the exact mechanism is not very clear.

Dysbiosis of the intestinal flora plays an important role in the pathogenesis of NEC, and its metabolite, as an intermediate bridge, has an effect on many diseases caused by intestinal microflora disorders [16, 17]. Succinate can be produced by *Enterococcaceae* and *Escherichia‐Shigella* [18, 19], which play important roles in the pathophysiology of diseases such as mastitis [20], diabetes [21], atopic dermatitis [22], and obesity [23]. Our previous studies revealed that succinate levels in the feces of NEC infants and neonatal mice were significantly increased, and exogenous succinate supplementation in NEC mice led to an increase in inflammation and changes in macrophage polarization markers [1, 18]; however, the mechanism of macrophage polarization is not well understood.

Recent studies have shown that succinate may bind to succinate receptor 1 (SUCNR1) and then activate the PI3K/AKT pathway, which plays an important role in the development of sepsis [24], ulcerative colitis [25], and acute lung injury [26]; however, whether this pathway is involved in NEC pathogenesis is unclear. The purpose of this study was to investigate whether succinate promotes M1‐type macrophage polarization and participates in the pathogenesis of NEC by activating the PI3K/AKT pathway.

## 2 Materials and Methods

### 2.1 Reagents and Antibodies

Succinate was obtained from Sigma‐Aldrich (S9512, Shanghai, China). Lipopolysaccharide (LPS) from *Escherichia coli* 055:B5 was purchased from Sigma‐Aldrich (L2880, Shanghai, China). The following rabbit antibodies were used: anti‐β‐actin (700068, ZENBIO, Chengdu, Sichuan, China), anti‐iNOS (18985‐1‐AP, Proteintech, Wuhan, Hubei, China), anti‐Arg1 (GTX109242, GeneTex, Irvine, California, United States), anti‐phospho‐AKT (ab81283, Abcam, Cambridge, Cambridgeshire, England), anti‐AKT (ab179463, Abcam, Cambridge, Cambridgeshire, England), anti‐HIF1a (GTX127309, GeneTex, Irvine, California, United States), anti‐SUCNR1 (ab272856, Abcam, Cambridge, Cambridgeshire, England), anti‐SUCNR1 (NBP1‐00861, Novus, Littleton, Colorado, United States), anti‐F4/80 (GB11027, Servicebio, Wuhan, Hubei, China), anti‐CD86 (19589, CST, Danvers, Massachusetts, United States), and anti‐CD206 (24595, CST, Danvers, Massachusetts, United States). HRP‐conjugated goat anti‐rabbit antibodies were obtained from Nakasugi Jinqiao (ZB‐2301, Beijing, China). The PI3K/AKT pathway inhibitor LY294002 was purchased from MCE (HY‐10108, Monmouth Junction, New Jersey, United States).

### 2.2 Animals and NEC Induction

The Animal Ethics Committee of Chongqing Medical University approved all the experiments performed in this study (No. CHCMU‐IACUC20240111013). C57BL/6J wild‐type (WT) mice were purchased from the Animal Experiment Center of Chongqing Medical University and maintained by the Animal Research Platform of the Children's Hospital, Chongqing Medical University. All the mice were kept in an environment at 25°C–27°C with a 12‐h light/dark cycle. Both male and female mouse pups were used, along with littermate controls when possible. Ten‐day‐old C57BL/J6 mice were divided into different groups according to the protocol of the in‐vivo experiments.

Induction of NEC was carried out as previously described [18, 27]. Neonatal mice were housed in custom‐made incubators and given formula milk (2 g of Similac Advance in 10 mL of 33% Esbilac Puppy Milk Replacer). The formula milk was administered via gavage via a 1.9‐Fr silicone tube. The mice were fed by gavage five times each day, continuously for 4 days, from 8:00 a.m. to 12:00 p.m. The milk dose (30 μL/g) was calculated on the basis of the body weight of newborn mice. Newborn mice were placed in a custom‐designed hypoxia chamber at 10 L/min filled with 100% nitrogen and exposed to hypoxia for 90 s. After the valve was closed and the mice were removed, cold stimulation was immediately applied by placing them in a refrigerator at 4°C for 10 minutes. Hypoxia and cold stimulation were performed twice daily (at 10:00 a.m. and 10:00 p.m.).

### 2.3 Experimental Design

To explore whether succinate promotes intestinal macrophage polarization, the neonatal mice for the experiment were randomly divided into three groups: (1) the control group (Control) mice were fed by their mothers in a normal environment; (2) the NEC group (NEC) mice were fed with formula milk during the induction of NEC; and (3) the NEC+Succinate group (NEC+Succi) mice were fed with succinate‐containing formula milk during the induction of NEC, and the final concentration of succinate in the formula was 100 mmol/L^18^. In our previous studies, succinate clearly affected macrophage polarization markers when it was administered at a concentration of 100 mmol/L; therefore, we used this dose of succinate for our experiments [18]. To clarify whether succinate promotes macrophage polarization through SUCNR1, we used anti‐SUCNR1 (NBP1‐00861, Novus, Littleton, Colorado, United States) to neutralize SUCNR1 and to clarify whether succinate promotes macrophage polarization through the PI3K/AKT signaling pathway, we inhibited PI3K with LY294002 [28]. Therefore, the remaining experimental mice were randomly divided into two other groups: (4) the NEC+Succinate+anti‐SUCNR1 group (NEC+Succi+SUCNR1‐Ab) mice in the induced NEC model were injected intraperitoneally with anti‐SUCNR1 (2.5 μg/g, day 1 of modeling) and (5) the NEC+Succinate+LY294002 group (NEC+Succi+LY) mice in the induced NEC model were injected intraperitoneally with LY294002 (20 mg/kg/d).

### 2.4 Histological Evaluation

In each mouse group, ileal tissues (1 cm) were fixed overnight in 4% paraformaldehyde and embedded in paraffin. Subsequently, 4 μm sections were stained with hematoxylin–eosin (HE). Histopathological analysis was performed on the basis of the published NEC damage scoring system [29, 30]. The analysis was performed in a blinded manner by a pathologist. Histological changes were graded as follows: 0 (normal) indicated no damage; 1 (mild) indicated epithelial cell separation and/or slight separation of the submucosal and/or lamina propria; 2 (moderate) indicated moderate separation of the submucosa and/or lamina propria and/or edema within the submucosal and muscular layers; 3 (severe) indicated focal villous denudation and severe separation of the submucosa and/or lamina propria and/or severe edema of the submucosal and muscular layers; and 4 (necrosis) indicated transmural necrosis [29, 30]. Mice with scores exceeding 2 were considered to have NEC.

### 2.5 Cell Culture

Mouse mononuclear macrophage leukemia cells (RAW264.7, TCM‐C766), obtained from Haixing Biosciences Co. Ltd. (Suzhou, Jiangsu China), were cultured in Dulbecco's modified Eagle's medium (DMEM, Gibco, Grant Island, New York, United States) supplemented with 10% fetal bovine serum (FBS, Sigma, St. Louis, Missouri, United States), 100 U/mL penicillin, and 100 μg/mL streptomycin (Beyotime, Shanghai, China) at 37°C with 5% CO_2_ in a humidified incubator. The cells were passaged every 24–48 h via standard procedures.

### 2.6 Cell Viability Assay and Treatment

To evaluate cell viability, a Cell Counting Kit‐8 (CCK‐8) assay was performed according to the manufacturer's instructions. The cells (2 × 10^4^ cells/well) were seeded in 96‐well plates and incubated with serum‐containing medium for 24 h. Then, LPS (50 ng/mL) [31] was used to coculture the cells with succinate (0.5, 1, 5 or 10 mmol/L) for 24 h, and the positive control was treated with LPS for 24 h. Then, 10 μL of CCK‐8 reagent was added to each well, and the cells were incubated for an additional 1 h at 37°C. The cells were subjected to a CCK8 assay, and the absorbance at 450 nm was measured via a microplate reader (BioTek Synergy H1, Windsor, Vermont, USA). Cell viability was calculated as a percentage of that of the control cells. RAW264.7 cells were pretreated with specific pharmacological inhibitors (20 μmol/L, LY294002) or incubated with an anti‐SUCNR1 antibody (IgG, 5 μg/mL) [26] for 1 h prior to stimulation with succinate (1 mmol/L). Moreover, RAW264.7 cells were treated with LPS or LPS + succinate for 24 h.

### 2.7 Immunofluorescence

The 4 μm tissue sections were deparaffinized, rehydrated, and subjected to antigen retrieval using heat‐mediated treatment with 0.01 mol/L sodium citrate buffer at pH 6.0. After that, 3% bovine serum albumin (BSA) was added, and the samples were blocked for 30 min. Following cooling and washing with tris‐buffered saline with tween 20 (TBST), nonspecific binding was blocked with 10% normal goat serum for 30 min. The tissue sections were then incubated overnight at 4°C with the relevant primary antibodies and subsequently incubated at room temperature in the dark with a fluorescence‐conjugated secondary antibody for 1 h. The following primary antibodies and concentrations were used: F4/80 (1:6000), GPR91 (1:500, Novus), CD86 (1:1000), and CD206 (1:3000). After the tissue sections were washed, tyramide signal amplification (TSA) dye (AiFang Biotech, Changsha, Hunan, China) was applied. The sections were then washed three times with phosphate buffered saline (PBS), and 4 ', 6‐diamidino‐2‐phenylindole (DAPI) was added to stain the cell nuclei, which were subsequently incubated for 10 min. All stained sections were initially scanned via a Nikon fluorescence microscope (Eclipse 80i, Chiyoda Ward, Tokyo, Japan). Three nonoverlapping fields of view were subsequently randomly selected from the intestinal basal region of each section for quantitative analysis. Image analysis was performed via ImageJ software and its standardized plug‐in for measuring the coexpression ratio of the fluorescent signals. The obtained data were statistically processed and visualized via GraphPad Prism software, and statistical testing was performed via one‐way ANOVA followed by Bonferroni correction.

### 2.8 Enzyme‐Linked Immunosorbent Assay

Total protein was extracted from intestinal tissues via PBS supplemented with a protease inhibitor cocktail (Selleck, Shanghai, China). The frozen intestinal tissues were removed from the refrigerator at −80°C. 100 μL PBS containing the cocktail was added to 10 mg of intestinal tissue and homogenized with a low‐temperature grinder for 4 min. Then, the tissue homogenate was centrifuged at 4°C (12000 rpm, 15 min). The concentrations of CD86 and CD206 in the supernatant of mouse intestinal tissue were measured via mouse‐specific CD86 and CD206ELISA kits (JONLNBIO, Shanghai, China) and a BioTek Synergy H1 instrument (Windsor, Vermont, USA) following the manufacturer's directions.

### 2.9 Quantitative Real‐Time Polymerase Chain Reaction (qRT‐PCR)

Total RNA was extracted from the distal ileum via the SteadyPure Quick RNA Extraction Kit (AG21023, Changsha, Hunan, China). The purity of the RNA was quantified using a NanoDrop spectrophotometer (Thermo Fisher Scientific, Waltham, Massachusetts, USA), and RNA samples with acceptable OD260/280 values (1.8–2.2) and OD260/230 values (> 2.0) were selected. RNA was reverse‐transcribed into cDNA using an Evo M‐MLV RT Mix Tracking Kit (AG11734, Changsha, Hunan, China) with gDNA Clean for quantitative real‐time polymerase chain reaction (qRT‐PCR). The quantitative real‐time polymerase chain reaction (qRT‐PCR) protocol was performed via a SYBR Green Premix Pro Taq HS qPCR kit (AG11733, Changsha, Hunan, China). *Hprt1* was used as an internal control, and the relative expression of mRNAs (*iNOS*, *TNF‐α*, *Arg1*, and *IL‐10*) in intestinal tissue was determined using the 2^−ΔΔCt^ method. The sequences of the primers used are provided in Table 1.

**TABLE 1:** The primer sequences.

Gene	Direction	Primers
*Hprt1*	Forward	TCAGTCAACGGGGGACATAAA
	Reverse	GGGGCTGTACTGCTTAACCAG
*iNOS*	Forward	CCAAGCCCTCACCTACTTCC
	Reverse	CTCTGAGGGCTGACACAAGG
*Arg1*	Forward	CCACAGTCTGGCAGTTGGAAG
	Reverse	GGTTGTCAGGGGAGTGTTGATG
*TNF‐a*	Forward	AGGGTCTGGGCCATAGAACT
	Reverse	CCACCACGCTCTTCTGTCTAC
*IL‐10*	Forward	TTCTTTCAAACAAAGGACCAGC
	Reverse	GCAACCCAAGTAACCCTTAAAG

### 2.10 Western Blot Analysis

Total protein was extracted from mammalian cells and intestinal tissue via radio immunoprecipitation assay (RIPA) lysis buffer (Beyotime, Shanghai, China) supplemented with protease inhibitor cocktails (Selleck, Shanghai, China). Intestinal tissue was homogenized with a low‐temperature grinder for 4 min and centrifuged (12000 rpm, 15 min) to obtain the supernatant. The total protein concentration was determined via an enhanced BSA protein assay kit (Beyotime, Shanghai, China). The protein supernatant was combined with sodium dodecyl sulfate sample buffer (NCM Biotech, Suzhou, Jiangsu, China) at a 4:1 ratio and heated at 100°C for 10 min to achieve denaturation. The protein samples were separated using 10% SDS‒PAGE and subsequently transferred onto polyvinylidene fluoride (PVDF) membranes (Millipore, Burlington, Massachusetts, USA). The membranes were washed and blocked with NcmBlot blocking buffer (NCM Biotech, Suzhou, Jiangsu, China) at room temperature for 15 min and then incubated overnight at 4°C with specific antibodies against *β*‐actin (HRP‐conjugated, 1:10000), iNOS (1:1000), ARG1 (1:1000), SUCNR1 (1:1500, Abcam), *p*‐AKT (1:4000), and AKT (1:10000). After the membranes were washed with TBST, they were incubated with HRP‐conjugated secondary antibodies for 90 min. The membranes were thoroughly washed with TBST, and the bands were subsequently visualized via enhanced chemiluminescence (ECL, ZENBIO, Chengdu, Sichuan, China). Relative quantification of the levels of target proteins was performed using Image Lab and ImageJ software.

### 2.11 Statistical Analysis

GraphPad Prism 9.0 (San Diego, California, United States) was used for graphing and statistical analysis. Normally distributed data are expressed as the means ± SD, and significant differences between groups were analyzed by one‐way ANOVA with Bonferroni correction. For the statistical analysis of the mouse weight data, two‐way ANOVA was used. For the statistical analysis of the mouse survival curves, the log‐rank (Mantel–Cox) test was used. The median and interquartile range were used to describe skewed distribution data, and significant differences were determined using the Kruskal‒Wallis test. All analyses were two‐sided and performed at a significance level of *p* < 0.05.

## 3 Results

### 3.1 Succinate Aggravated Intestinal Damage and Disrupted the Proinflammatory and Anti‐Inflammatory Balance in NEC Neonatal Mice

**FIGURE 1:**
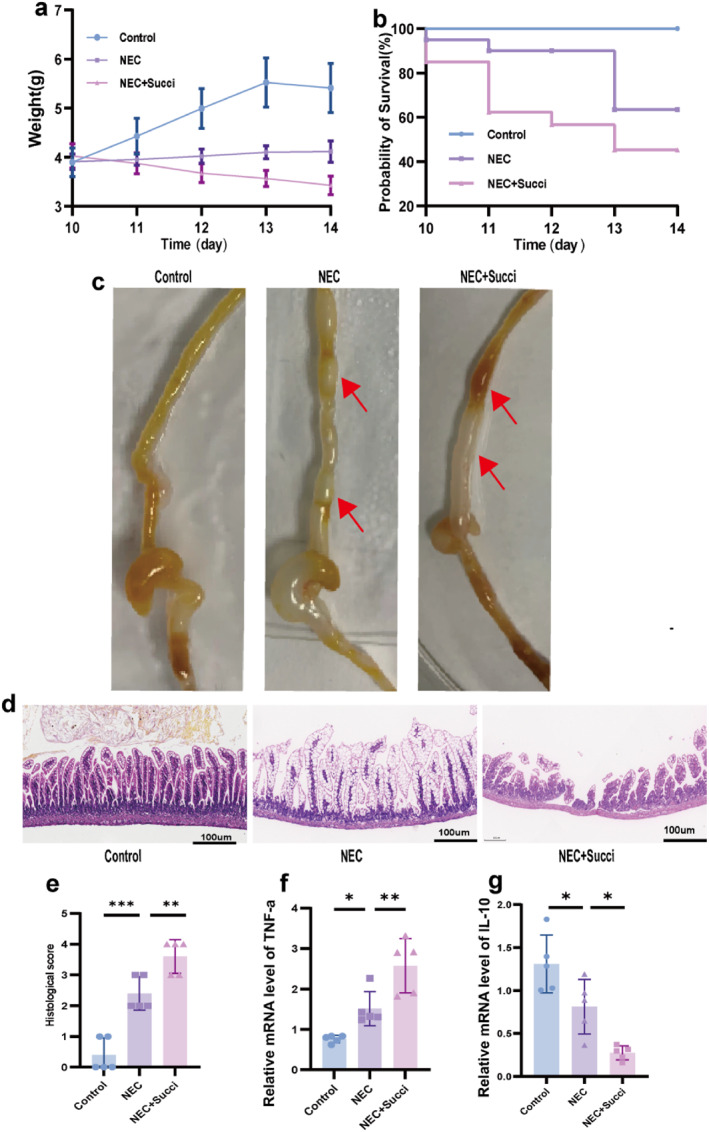
Succinate exacerbated the severity of NEC in mice. (a) Body weight changed in neonatal mice among the three groups (*n* = 8). Statistical analysis was performed using two‐way ANOVA with multiple comparisons. (b) Survival curves of neonatal mice in the three groups. Each group initially contained 20 mice. Statistical analysis: log‐rank (Mantel–Cox) test (*p* < 0.05). (c) Gross morphology of the intestinal tissues of neonatal mice in the three groups. Red arrows indicate gas accumulation or bleeding. (d) Representative photomicrographs of terminal ileal sections from each of the three groups. Magnification: 100x. (e) Histological scores of intestinal regions in the three groups (*n* = 5). 95% confidence intervals: Control versus NEC (1.1330, 2.8670) and NEC versus NEC+Succi (−2.0670, −0.3331). Statistical analysis: Kruskal–Wallis test. The relative mRNA expression of *TNF‐a* (f) and *IL‐10* (g) in the control, NEC, and NEC+Succi groups (*n* = 5). 95% confidence intervals of *TNF‐a*: Control versus NEC (−1.497, −0.0016) and NEC versus NEC+Succi (−1.809, −0.3133); 95% confidence intervals of *IL‐10*: Control versus NEC (0.05760, 0.9341) and NEC versus NEC+Succi (0.1000, 0.9765). The data are presented as the mean ± SD. Statistical significance was assessed with one‐way ANOVA followed by Bonferroni correction. *: *p* < 0.05, **: *p* < 0.01, ***: *p* < 0.001, and ****: *p* < 0.0001. Control: control group, NEC: necrotizing enterocolitis group, and NEC + Succi: necrotizing enterocolitis + succinate group.

During the NEC modeling period, the control group exhibited optimal weight gain, whereas the NEC group demonstrated a slower rate of weight gain. In contrast, the NEC+Succi group experienced significant weight loss (Figure 1a). During the induction of NEC, there were no deaths in the control group, and the mortality rates in the NEC group and the NEC+Succi group were 35% and 50%, respectively. The survival curves of the three groups were significantly different (*χ* ^2^ = 15.20 and *p* < 0.05) (Figure 1b). Naked‐eye observation showed (Figure 1c) that there was no damage to the intestinal tract in the control group, whereas the NEC group presented significant intestinal tissue gas accumulation, intestinal wall thinning, and bead‐like changes. In addition to these changes associated with NEC, notable bleeding and necrosis were observed in the NEC+Succi group. Histological analysis (Figure 1d,e) revealed that the NEC+Succi group presented the most severe structural damage to intestinal tissue, with significantly higher histologic damage scores than the other groups. Compared with the NEC+Succi group, the NEC group showed relatively less intestinal tissue damage and significantly lower histologic scores. Statistical analysis showed that the difference between the groups was significant (*p* < 0.05). In addition, qRT‐PCR experiments were performed to determine the levels of the proinflammatory factor tumor necrosis factor‐alpha (*TNF‐α*) and the anti‐inflammatory cytokine interleukin‐10 (*IL‐10*) in intestinal tissues. Compared with the control group, the NEC group presented higher levels of *TNF‐α* (Figure 1f) and lower levels of *IL‐10* (Figure 1g). Furthermore, the NEC+Succi group showed even higher *TNF‐α* levels and lower *IL‐10* levels compared to the NEC group (*p* < 0.05). These findings suggest that exogenous administration of succinate may exacerbate intestinal damage in mice and further disrupt the balance between proinflammatory and anti‐inflammatory responses.

### 3.2 Effects of Succinate Administration on the Viability of LPS‐Induced Mononuclear Macrophage Leukemia Cells (RAW264.7 Cells)

**FIGURE 2:**
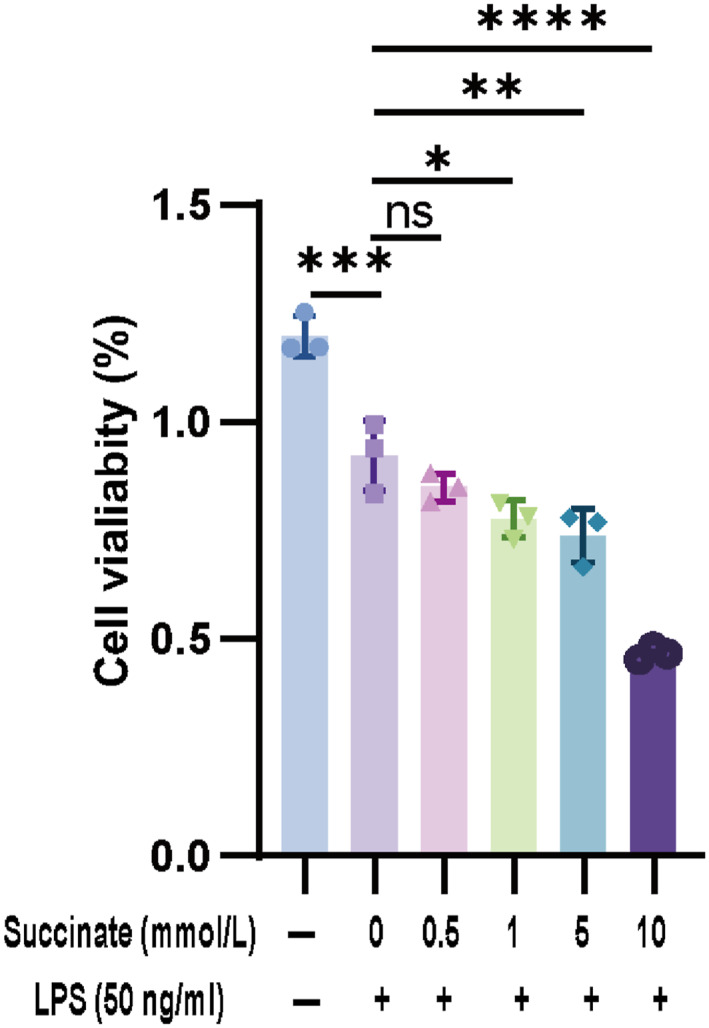
RAW264.7 cells were treated with different concentrations of succinate for 24 h.

To determine the optimal concentration of succinate to administer to mouse RAW264.7, we investigated the effects of succinate at concentrations of 0.5, 1, 5, and 10 mmol/L on RAW264.7 cells. Compared with that of the control group, the viability of the LPS‐induced RAW264.7 cells decreased (Figure 2). Furthermore, all concentrations of succinate except 0.5 mmol/L significantly aggravated LPS‐induced injury in RAW264.7 cells (*p* < 0.05). Based on these results, a low dose of succinate (1 mmol/L) was selected for subsequent experiments.

Cell viability was determined using a CCK‐8 assay (*n* = 3). 95% confidence intervals: Control versus LPS (0.1494, 0.4028), LPS versus LPS+Succi (0.5 mmol/L, −0.0539, 0.1995), LPS versus LPS+Succi (1 mmol/L, 0.0194, 0.2728), LPS versus LPS+Succi (5 mmol/L, 0.0572, 0.3106), and LPS versus LPS+Succi (10 mmol/L, 0.3294, 0.5828). The data are presented as the mean ± SD. Statistical significance was assessed with one‐way ANOVA followed by Bonferroni correction. *: *p* < 0.05, **: *p* < 0.01, ***: *p* < 0.001, and ****: *p* < 0.0001. Control: control group; LPS: lipopolysaccharide group; LPS + Succi: lipopolysaccharide + succinate group.

### 3.3 Succiniate Aggravated LPS‐Induced M1 Polarization in Raw264.7 Cells

**FIGURE 3:**
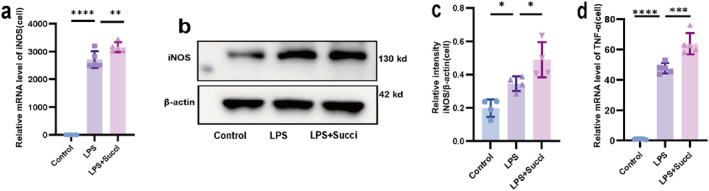
Succinate aggravated LPS‐induced M1 polarization in RAW264.7 cells. (a) The relative mRNA expression of *iNOS* in the control, LPS, and LPS+Succi groups was determined using qRT‐PCR (*n* = 5). 95% confidence intervals: Control versus LPS (−3041, −2378) and LPS versus LPS+Succi (−775.4, −112.8). (b, c) The relative protein expression of iNOS in the control, LPS, and LPS+Succi groups was determined using western blotting (*n* = 4). 95% confidence intervals: Control versus LPS (−0.2865, −0.0077), LPS versus LPS+Succi (−0.2823, −0.0035). (d) The relative mRNA expression of *TNF‐α* in the control, LPS, and LPS+Succi groups was determined using qRT‐PCR (*n* = 5). 95% confidence intervals: Control versus LPS (−54.04, −39.31) and LPS versus LPS+Succi (−23.69, −8.962). The data are presented as the mean ± SD. Statistical significance was assessed with one‐way ANOVA followed by Bonferroni correction. *: *p* < 0.05, **: *p* < 0.01, ***: *p* < 0.001, and ****: *p* < 0.0001. Control: control group; LPS: lipopolysaccharide group; LPS + Succi: lipopolysaccharide + succinate group.

To investigate whether succinate promotes M1 polarization in RAW264.7 cells. We detected the mRNA expression of *iNOS*, a marker of M1 macrophages, by qRT‐PCR and measured its protein expression by western blot. The mRNA expression of the proinflammatory factor *TNF‐α* was also assessed by qRT‐PCR. The results showed that after LPS treatment, the mRNA expression level of *iNOS* (Figure 3a) in LPS group was significantly higher than that in the control group (*p* < 0.05). Moreover, the *iNOS* mRNA level in the LPS+Succi group was higher than that in the LPS group (*p* < 0.05). The changes in iNOS protein levels were consistent with the trends in its mRNA expression (Figure 3b,c). Meanwhile, the mRNA levels of *TNF‐α*, a proinflammatory factor, showed the same trend as those of *iNOS* in all groups (*p* < 0.05; Figure 3d). These results suggest that succinate may exacerbate the LPS‐induced polarization of RAW264.7 cells toward the M1 phenotype and promote their secretion of proinflammatory factors.

### 3.4 Succinate Promoted the Polarization of NEC Intestinal Macrophages Toward the M1 Phenotype

**FIGURE 4:**
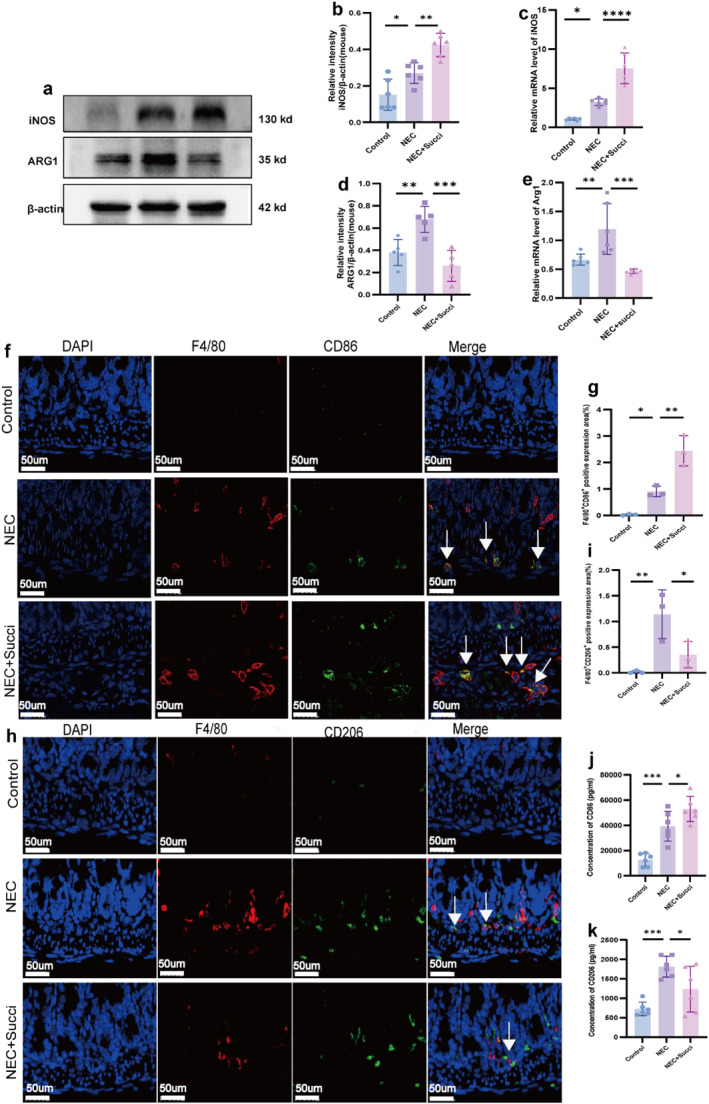
Succinate promoted the polarization of NEC intestinal macrophages toward the M1 phenotype. (a, b, and d) The relative protein expressions of iNOS and ARG1 in the control, NEC, and NEC+Succi groups were determined using western blot (*n* = 5–6/group). 95% confidence intervals of iNOS: Control versus NEC (−0.2186, −0.0175) and NEC versus NEC+Succi (−0.2548, −0.05374). 95% confidence intervals of ARG1: Control versus NEC (−0.5004, −0.09522) and NEC versus NEC+Succi (0.2153, 0.6204). (c, e) The relative mRNA expression levels of *iNOS* and *Arg1* in the control, NEC, and NEC+Succi groups were determined using qRT‐PCR (*n* = 6). 95% confidence intervals of *iNOS*: Control versus NEC (−3.855, −0.4817) and NEC versus NEC+Succi (−6.012, −2.638). 95% confidence intervals of *Arg1*: Control versus NEC (−0.9066, −0.1555) and NEC versus NEC+succi (0.3561, 1.107). The expression levels of the macrophage markers F4/80^+^/CD86^+^(f, g) and F4/80^+^/CD206^+^(h, i) were assessed in the guts of pups by immunofluorescence; the arrows indicate coexpression (*n* = 3); and the data are representative of 3 fields/samples. Scale bars = 50 μm. 95% confidence intervals of F4/80^+^/CD86^+^: Control versus NEC (−1.731, −0.02509) and NEC versus NEC+Succi (−2.395, −0.6887); 95% confidence intervals of F4/80^+^/CD206^+^: Control versus NEC (−1.881, −0.3692) and NEC versus NEC+Succi (0.03353, 1.545). The relative protein expressions of CD86 (j) and CD206 (k) in the gut of pups were determined using ELISA (*n* = 6). 95% confidence intervals of CD86: Control versus NEC (−40196, −13069) and NEC versus NEC+Succi (−27210, −82.99); 95% confidence intervals of CD206: Control versus NEC (−1642, −533.5) and NEC versus NEC+Succi (26.92, 1135). The data are expressed as the mean ± SD. Statistical significance was assessed with one‐way ANOVA followed by Bonferroni correction. *: *p* < 0.05, **: *p* < 0.01, ***: *p* < 0.001, and ****: *p* < 0.0001. Control: control group, NEC: necrotizing enterocolitis group, and NEC + Succi: necrotizing enterocolitis + succinate group.

To explore whether succinate promotes the polarization of NEC intestinal macrophages toward the M1 phenotype, we also assessed the expression characteristics of M1 and M2 macrophage phenotype markers in vivo. The results showed that the protein expression level of the M1 macrophage marker iNOS was higher in the NEC group than in the control group (*p* < 0.05 and Figure 4a,b). The iNOS protein expression was further upregulated after succinate addition to the NEC group (*p* < 0.05). Correspondingly, the expression trend of *iNOS* mRNA was consistent with its protein level (*p* < 0.05 and Figure 4c). For the M2 macrophage marker ARG1, its protein expression was higher in the NEC group than in the control group (*p* < 0.05 and Figure 4a,d) but decreased significantly after succinate treatment (*p* < 0.05). In addition, the mRNA expression trend of *Arg1* was also consistent with the changes in its protein levels (*p* < 0.05, Figure 4e). Immunofluorescence double‐labeling confirmed that F4/80^+^/CD86^+^ (Figure 4f,g) coexpression (type M1) was increased in the intestinal basal layer of the NEC group compared with that in the control group (*p* < 0.05), and the proportion of this colocalization continued to increase after the addition of succinate to the NEC group (*p* < 0.05), whereas although F4/80^+^/CD206^+^ (Figure 4h,i) coexpression (type M2) was elevated in the intestinal basal layer of the NEC group, its colocalization level could be decreased by the addition of succinate to the NEC group (*p* < 0.05). The ELISA results further confirmed that the changes in the protein levels of CD86 (Figure 4j) and CD206 (Figure 4k) were consistent with the above findings. These results suggest that exogenous succinate intervention may promote the polarization of NEC intestinal macrophages toward the M1 phenotype.

### 3.5 Succinate Promoted Macrophage Polarization via SUCNR1 in Vitro

**FIGURE 5:**
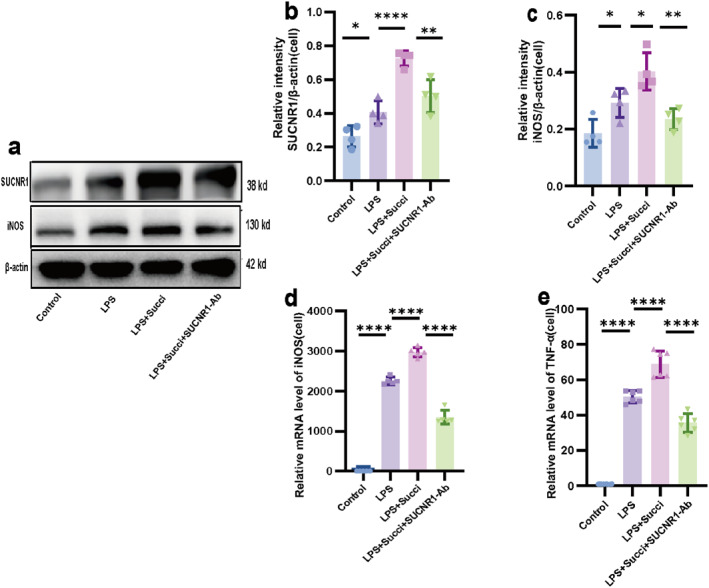
Succinate promoted macrophage polarization via SUCNR1 in vitro. The relative protein expression levels of SUCNR1 (a, b) and iNOS (a, c) in the control, LPS, LPS+Succi, and LPS+Succi+SUCNR1‐Ab groups were determined using western blot (*n* = 4–5). 95% confidence intervals of SUCNR1: Control versus LPS (−0.2797, −0.0045), LPS versus LPS+Succi (−0.4575, −0.1823), and LPS+Succi versus LPS+Succi+SUCNR1‐Ab (0.0858, 0.3610); 95% confidence intervals of iNOS: Control versus LPS (−0.2079, −0.0051), LPS versus LPS+Succi (−0.2113, −0.0085), and LPS+Succi versus LPS+Succi+SUCNR1‐Ab (0.06520, 0.2680). The relative mRNA expressions of *iNOS* (d) and *TNF‐α* (e) in the control, LPS, LPS+Succi, and LPS+Succi+SUCNR1‐Ab groups were determined using qRT‐PCR (*n* = 5–6). 95% confidence intervals of *iNOS*: Control versus LPS (−2447, −2058), LPS versus LPS+Succi (−906.5, −517.1), and LPS+Succi versus LPS+Succi+SUCNR1‐Ab (1425, 1815); 95% confidence intervals of *TNF‐α*: Control versus LPS (−56.74, −42.11), LPS versus LPS+Succi (−25.73, −11.10), and LPS+Succi versus LPS+Succi+SUCNR1‐Ab (25.97, 40.60). The data are expressed as the mean ± SD. Statistical significance was assessed with one‐way ANOVA followed by Bonferroni correction. *: *p* < 0.05, **: *p* < 0.01, ***: *p* < 0.001, and ****: *p* < 0.0001. Control: control group, LPS: lipopolysaccharide group, LPS + Succi: lipopolysaccharide + succinate group, and LPS + Succi + SUCNR1‐Ab: lipopolysaccharide + succinate + anti‐SUCNR1 group.

To investigate whether succinate facilitates macrophage polarization through SUCNR1. We used western blot to detect the protein expression of SUCNR1 and the M1 macrophage marker iNOS in RAW264.7 cells and combined it with qRT‐PCR to analyze the mRNA levels of *iNOS* and the proinflammatory factor *TNF‐α*. Compared with the control group, LPS stimulation upregulated the protein expression of SUCNR1 in RAW264.7 cells (*p* < 0.05; Figure 5a,b). Compared with that in the LPS group, the expression of SUCNR1 was further increased in the LPS+Succi group (*p* < 0.05); after the addition of the SUCNR1 neutralizing antibody, the expression of this receptor was significantly inhibited (*p* < 0.05). Notably, the trend of iNOS protein expression was consistent with that of SUCNR1 (Figure 5a,c). The qRT‐PCR results further revealed that, compared with the LPS+Succi group, SUCNR1 neutralizing antibody intervention not only reduced the *iNOS* mRNA level (*p* < 0.05; Figure 5d) but also effectively suppressed the expression of the downstream inflammatory factor *TNF‐α* (*p* < 0.05; Figure 5e). These results showed that succinate may promote M1 polarization of macrophages through SUCNR1 in vitro.

### 3.6 Succinate Promoted NEC Intestinal Macrophage Polarization via SUCNR1

**FIGURE 6:**
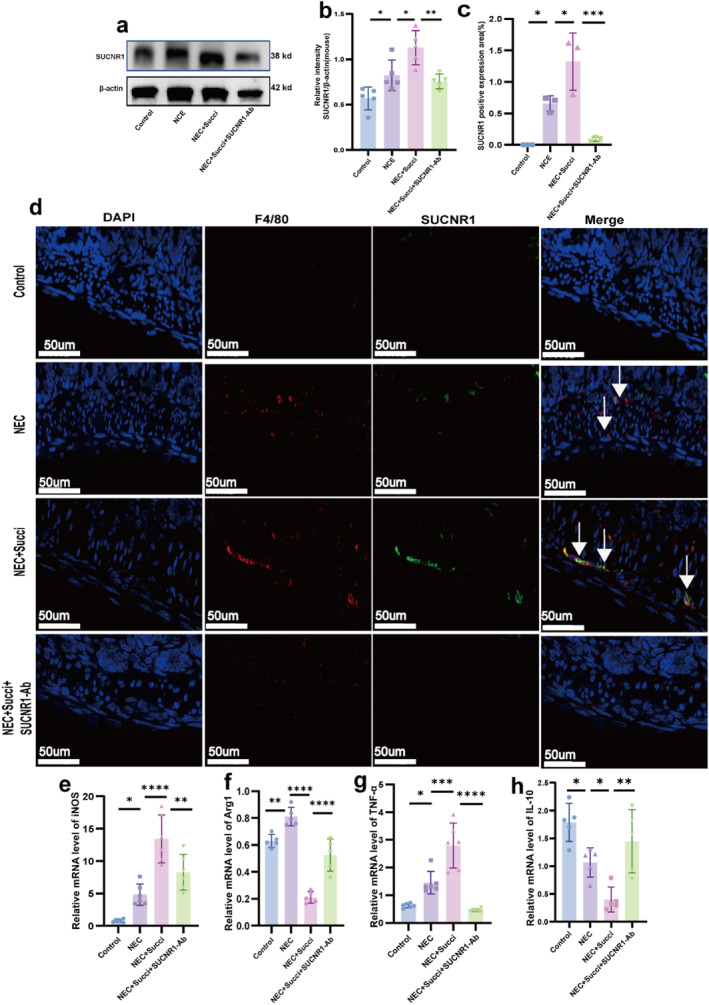
Succinate promoted NEC intestinal macrophage polarization via SUCNR1. The relative protein expression of SUCNR1 (a, b) in the control, NEC, NEC+Succi, and NEC+Succi+SUCNR1‐Ab groups was determined via western blotting. 95% confidence intervals: Control versus NEC (−0.5035, −0.005196), NEC versus NEC+Succi (−0.5533, −0.0550), and NEC+Succi versus NEC+Succi+SUCNR1‐Ab (0.1235, 0.6218). Coexpression of the macrophage markers F4/80 and SUCNR1 (c, d) was assessed in the four groups by immunofluorescence staining. The arrows indicate coexpression (*n* = 3), and the data are representative of 3 fields/samples. Scale bars = 50 μm. 95% confidence intervals: Control versus NEC (−1.235, −0.07313), NEC versus NEC+Succi (−1.249, −0.08764), and NEC+Succi versus NEC+Succi+SUCNR1‐Ab (0.6536, 1.815). The relative mRNA expression of *iNOS* (e), *Arg1* (f), *TNF‐α* (g), and *IL‐10* (h) in the four groups was determined using qRT‐PCR (*n* = 5–6). 95% confidence intervals of *iNOS*: Control versus NEC (−7.706, −0.3161), NEC versus NEC+Succi (−12.31, −4.917), and NEC+Succi versus NEC+Succi+SUCNR1‐Ab (1.469, 8.858). 95% confidence intervals of *Arg1*: Control versus NEC (−0.3114, −0.05325), NEC versus NEC+Succi (0.4717, 0.7298), NEC+Succi versus NEC+Succi+SUCNR1‐Ab (−0.4429, −0.1847). 95% confidence intervals of *TNF‐α*: Control versus NEC (−1.515, −0.1381), NEC versus NEC+Succi (−2.030, −0.6538), and NEC+Succi versus NEC+Succi+SUCNR1‐Ab (1.652, 3.028). 95% confidence intervals of *IL‐10*: Control versus NEC (−1.911, −0.5409), NEC versus NEC+Succi (1.058, 2.429), and NEC+Succi versus NEC+Succi+SUCNR1‐Ab (−1.783, −0.4124). The data are expressed as the mean ± SD. Statistical significance was assessed with one‐way ANOVA followed by Bonferroni correction. *: *p* < 0.05, **: *p* < 0.01, ***: *p* < 0.001, and ****: *p* < 0.0001. Control: control group, NEC: necrotizing enterocolitis group, NEC + Succi: necrotizing enterocolitis + succinate group, and NEC + Succi + SUCNR‐Ab: necrotizing enterocolitis + succinate + anti‐SUCNR1 group.

To clarify whether succinate facilitates the polarization of NEC intestinal macrophages through SUCNR1, we detected the protein expression of SUCNR1 by western blot, observed the coexpression of the intestinal macrophage marker F4/80 with SUCNR1 by immunofluorescence, and analyzed the macrophage markers (*iNOS* and *Arg1*) and inflammatory factors (*TNF‐α* and *IL‐10*) via qRT‒PCR. The western blot results revealed that SUCNR1 expression was significantly upregulated in the NEC group compared with the control group (*p* < 0.05), and SUCNR1 expression was further increased in the NEC+Succi group (*p* < 0.05), whereas SUCNR1‐neutralizing antibody treatment reversed this effect (*p* < 0.05; Figure 6a,b). The immunofluorescence results revealed that in the NEC intestinal basal layer and its surroundings, SUCNR1‐positive cells colocalized with basal layer macrophages. Compared with those in the control group, the proportions of F4/80 and SUCNR1 coexpressed cells were greater in the NEC group (*p* < 0.05). The NEC+Succi group presented an even greater increase in F4/80 and SUCNR1 colocalized cells than the NEC group (*p* < 0.05); however, the proportion was significantly lower in the NEC+Succi+SUCNR1‐Ab group than in that of the NEC+Succi group (*p* < 0.05; Figure 6c,d). Compared with the NEC+Succi group, the NEC+Succi+SUCNR1‐Ab group presented a synchronized downregulation of the M1‐type marker *iNOS* (Figure 6e) and the proinflammatory factor *TNF‐α* (Figure 6g), accompanied by a rebound in the expression of the M2‐type marker *Arg1* (Figure 6f) and the anti‐inflammatory factor *IL‐10* (Figure 6h), compared with the NEC+Succi group (*p* < 0.05). These results indicated that succinate may promote NEC intestinal macrophage polarization via SUCNR1 in vivo.

### 3.7 The PI3K/AKT Pathway Participated in Succinate‐Promoted Macrophage Polarization in Vitro

**FIGURE 7:**
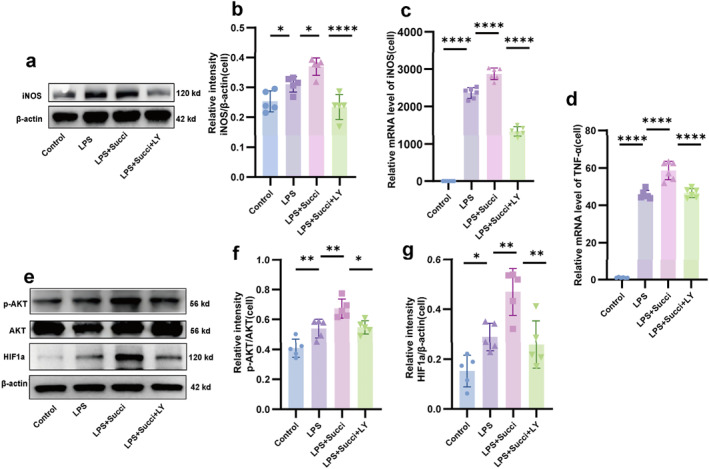
The PI3K/AKT pathway participated in succinate‐promoted macrophage polarization in vitro. The protein expression level of iNOS (a, b) in the four groups (*n* = 5) was measured by western blot. 95% confidence intervals: Control versus LPS (−0.1134, −5.074e‐005), LPS versus LPS+Succi (−0.1163, −0.0029), and LPS+Succi versus LPS+Succi+LY (0.07872, 0.1921). The mRNA expressions of *iNOS* (c) and *TNF‐α* (d) in the four groups (*n* = 6) were evaluated by qRT‒PCR. 95% confidence intervals of *iNOS*: Control versus LPS (−2543, −2173), LPS versus LPS+Succi (−699.7, −328.9), and LPS+Succi versus LPS+Succi+LY (1355, 1726). 95% confidence intervals of *TNF‐a*: Control versus LPS (−49.12, −40.14), LPS versus LPS+Succi (−17.42, −8.445), and LPS+Succi versus LPS+Succi+LY (7.640, 16.62). Protein expression levels of *p*‐AKT (e, f) and HIF1a (e, g) in the four groups (*n* = 5) were determined using Western blot. 95% confidence intervals of *p*‐AKT: Control versus LPS (−0.2298, −0.03228), LPS versus LPS+Succi (−0.2324, −0.0348), and LPS+Succi versus LPS+Succi+LY (0.0266, 0.2241); 95% confidence intervals of HIF1a: Control versus LPS (−0.2696, −0.0031), LPS versus LPS+Succi (−0.3139, −0.04737), and LPS+Succi versus LPS+Succi+LY (0.0776, 0.3441). The data are expressed as the mean ± SD. Statistical significance was assessed with one‐way ANOVA followed by Bonferroni correction. *: *p* < 0.05, **: *p* < 0.01, and ****: *p* < 0.0001. Control: control group, LPS: lipopolysaccharide group, LPS + Succi: lipopolysaccharide + succinate group, and LPS + Succi + LY: lipopolysaccharide + succinate + LY294002 group.

To elucidate whether the PI3K/AKT pathway is involved in succinate‐promoted macrophage polarization, after the PI3K signal was inhibited with LY294002, we detected the protein expression of the M1‐type macrophage marker iNOS in RAW264.7 cells by western blot and analyzed the changes in the mRNA levels of *iNOS* and the proinflammatory factor *TNF‐α* by qRT‐PCR. Western blot results showed that iNOS protein expression level in the LPS group was higher than that in the control group (*p* < 0.05). Succinate treatment further promoted the LPS‐induced upregulation of iNOS protein (*p* < 0.05), whereas LY294002 significantly inhibited this upregulation (*p* < 0.05; Figure 7a,b). Furthermore, qRT‐PCR results showed that the expression trends of both *iNOS* mRNA (Figure 7c) and *TNF‐α* mRNA (Figure 7d) in each group were consistent with the protein levels of iNOS, and all differences were statistically significant (*p* < 0.05).

We also used western blot to evaluate the expression of PI3K/AKT pathway proteins in RAW264.7 cells. The results showed that the expression of *p*‐AKT (Figure 7e,f) and HIF1a (Figure 7e,g) in the LPS‐treated group were higher than that in the control group (*p* < 0.05), and supplementing the LPS group with succinate significantly increased the expression of *p*‐AKT and HIF1a (*p* < 0.05). Additionally, the expression levels of *p*‐AKT and HIF1a were obviously decreased in the LPS+Succi+LY group (*p* < 0.05). These results suggest that succinate may participate in the polarization of macrophages through the PI3K/AKT pathway in vitro.

### 3.8 The PI3K/AKT Pathway Participated in Succinate‐Promoted Macrophage Polarization in Vivo

**FIGURE 8:**
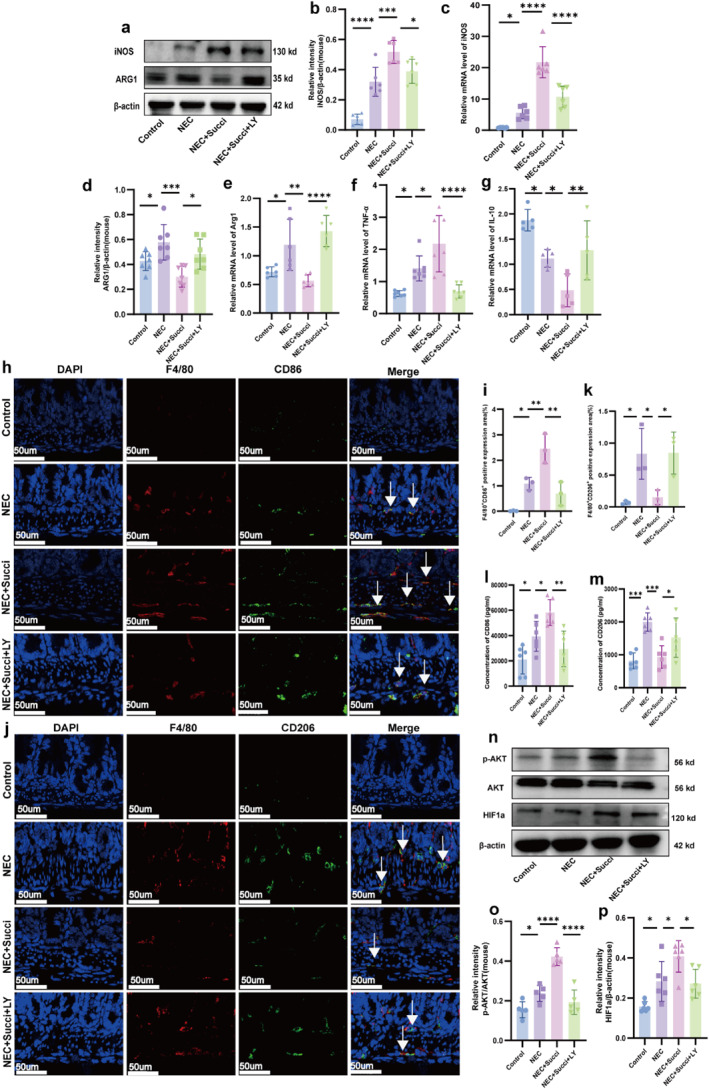
The PI3K/AKT pathway participated in succinate‐promoted macrophage polarization in vivo. Protein expression levels of iNOS (a, b) and ARG1 (a, d) in the four groups (*n* = 5–7) were determined using western blotting. 95% confidence intervals of iNOS: Control versus NEC (−0.3635, −0.1355), NEC versus NEC+Succi (−0.3118, −0.08381), and NEC+Succi versus NEC+Succi+LY (0.0150, 0.2430). 95% confidence intervals of ARG1: Control versus NEC (−0.3017, −0.0016), NEC versus NEC+Succi (0.1268, 0.427), and NEC+Succi versus NEC+Succi+LY (−0.3313, 0.0311). The relative mRNA expression levels of *iNOS* (c), *Arg1* (e), *TNF‐α* (f), and *IL‐10* (g) in the four groups were measured via qRT‐PCR (*n* = 5–7). 95% confidence intervals of *iNOS*: Control versus NEC (−9.546, −0.0024), NEC versus NEC+Succi (−20.93, −11.39), and NEC+Succi versus NEC+Succi+LY (6.257, 15.80). 95% confidence intervals of *Arg1*: Control versus NEC (−0.8787, −0.0653), NEC versus NEC+Succi (0.2198, 1.033), and NEC+Succi versus NEC+Succi+LY (−1.273, −0.4600). 95% confidence intervals of *TNF‐α*: Control versus NEC (−1.459, −0.1022), NEC versus NEC+Succi (−1.445, −0.0879), and NEC+Succi versus NEC+Succi+LY (0.8026, 2.159). 95% confidence intervals of *IL‐10*: Conrol versus NEC (−4.311, −0.5596), NEC versus NEC+Succi (0.4151, 4.167), and NEC+Succi versus NEC+Succi+LY (−3.802, −0.05027). Coexpression of macrophage markers F4/80^+^/CD86^+^ (h, i) and F4/80^+^/CD206^+^ (j, k) was assessed in the four groups by immunofluorescence (*n* = 3), and the arrows indicate coexpression. The data are representative of 3 fields/samples; scale bars = 50 μm. 95% confidence intervals of F4/80^+^/CD86^+^: Control versus NEC (−2.030, −0.1076), NEC versus NEC+Succi (−2.328, −0.4062), and NEC+Succi versus NEC+Succi+LY (0.7982, 2.720). 95% confidence intervals of F4/80^+^/CD206^+^: Control versus NEC (−1.416, −0.1123), NEC versus NEC+Succi (0.0283, 1.332), and NEC+Succi versus NEC+Succi+LY (−1.343, −0.04030). The relative protein expression of CD86 (L) and CD206 (m) in the four groups was determined using ELISA (*n* = 6). 95% confidence intervals of CD86: Control versus NEC (−36445, −227.1), NEC versus NEC+Succi (−37091, −873.8), and NEC+Succi versus NEC+Succi+LY (10741, 46959). 95% confidence intervals of CD206: Control versus NEC (−1771, −589.1), NEC versus NEC+Succi (473.5, 1655), and NEC+Succi versus NEC+Succi+LY (−1183, −1.631). Protein expression levels of *p*‐AKT (n, o) and HIF1a (n, p) in the four groups (*n* = 5–6) were determined via western blotting. 95% confidence intervals of *p*‐AKT: Control versus NEC (−0.1631,‐0.0021), NEC versus NEC+Succi (−0.2660, −0.1051), and NEC+Succi versus NEC+Succi+LY (0.1498, 0.3107). 95% confidence intervals of HIF1a: Control versus NEC (−0.2375, −0.01392), NEC versus NEC+Succi (−0.2370, −0.01352), and NEC+Succi versus NEC+Succi+LY (0.0249, 0.2484). The data are expressed as the mean ± SD. Statistical significance was assessed with one‐way ANOVA followed by Bonferroni correction. *: *p* < 0.05, **: *p* < 0.01, ***P: < 0.001, and ****: *p* < 0.0001. Control: control group, NEC: necrotizing enterocolitis group, NEC + Succi: necrotizing enterocolitis + succinate group, and NEC + Succi + LY: necrotizing enterocolitis + succinate + LY294002 group.

To investigate whether succinate promotes the polarization of intestinal macrophages in NEC through the PI3K/AKT pathway, we evaluated the expression of macrophage markers and inflammatory factors using qRT‐PCR, western blot, immunofluorescence, and ELISA following the administration of the PI3K inhibitor LY294002 in vivo. The results showed that compared to the control group, the protein expression level of the M1 macrophage marker iNOS was increased in the NEC group (*p* < 0.05). The addition of succinate to the NEC group further upregulated iNOS protein expression (*p* < 0.05), whereas LY294002 treatment partially reversed this effect (*p* < 0.05 and Figure 8 a,b). The expression trend of *iNOS* mRNA in each group was consistent with its protein levels (*p* < 0.05 and Figure 8c). In contrast, the protein level of the M2 macrophage marker ARG1 was also higher in the NEC group than in the control group (*p* < 0.05). However, its expression was reduced in the NEC + Succi group (*p* < 0.05), and this decrease was partially reversed by LY294002 treatment (*p* < 0.05, Figure 8a,d). The mRNA expression level of *Arg1* mirrored its protein levels across all groups (*p* < 0.05 and Figure 8e). Furthermore, after LY294002 treatment, the NEC + Succi group exhibited a significant decrease in the mRNA expression of the proinflammatory cytokine *TNF‐α* (*p* < 0.05 and Figure 8f), along with a significant increase in the mRNA expression of the anti‐inflammatory cytokine *IL‐10* (*p* < 0.05 and Figure 8g). Immunofluorescence analysis revealed that the coexpression level of F4/80^+^/CD86^+^ cells in the NEC group was greater than that in the control group (*p* < 0.05), and F4/80^+^/CD86^+^ expression was significantly increased by succinate supplementation in the NEC group, whereas LY294002 treatment reduced this effect (*p* < 0.05; Figure 8h,i). Moreover, the colocalization of F4/80^+^/CD206^+^ cells in the gut of NEC mice was higher than that in the control group (*p* < 0.05), but the coexpression of these cells was suppressed by succinate supplementation in the NEC group (*p* < 0.05), while treatment with the PI3K inhibitor LY294002 partially restored the expression (*p* < 0.05 and Figure 8j,k). Similarly, the trend of the ELISA results for the protein expression of CD86 (Figure 8l) and CD206 (Figure 8m) was consistent with the immunofluorescence results (*p* < 0.05).

We also measured the expression of PI3K/AKT pathway proteins in vivo via western blot. The results showed that the protein expressions of both *p*‐AKT and HIF1a were higher in the NEC group than those in the control group (*p* < 0.05), whereas supplementation of the NEC group with succinate significantly increased the expression of *p*‐AKT and HIF1a (*p* < 0.05); however, their expression levels obviously decreased in the NEC+Succi+LY group (*p* < 0.05 and Figure 8n–p). Therefore, both in vitro and in vivo studies have suggested that succinate may participate in the polarization of macrophages through the PI3K/AKT pathway.

## 4 Discussion

This study investigated the role of succinate in NEC intestinal macrophage polarization. Our results showed that succinate exacerbated intestinal tissue damage in NEC, induced an imbalance between proinflammatory and anti‐inflammatory responses, and promoted the polarization of macrophages toward the M1 phenotype. The polarization effect of succinate on NEC macrophages was partially mediated by SUCNR1. The results indicated that the polarization of M1 macrophages was inhibited in both SUCNR1‐blocked mice and Raw264.7 cells. Mechanistically, the PI3K/AKT pathway is involved in the succinate‐induced polarization of NEC intestinal macrophages.

We found that succinate exacerbated the severity of NEC in mice and resulted in an imbalance between proinflammatory and anti‐inflammatory responses. This was evidenced by increased intestinal tissue damage, increased mortality, weight loss, and upregulation of the expression of the proinflammatory factor *TNF‐α* and downregulation of the expression of the anti‐inflammatory factor *IL‐10* in mice. Succinate is an intermediate metabolite or end‐product of many gut microbes and accumulates abnormally when there is an imbalance between succinate‐producing and succinate‐consuming *Bacteriaceae* [18, 32]. In the intestinal lumen and feces, succinate concentrations typically range from 1 to 3 mmol/kg [33]. Studies have shown that exogenous succinate supplementation promotes disease progression by inducing body weight loss, shortening colon length, increasing tissue damage scores, and proinflammatory/anti‐inflammatory homeostatic imbalance in mice with inflammatory bowel disease (IBD) [33, 34]. These findings are consistent with our observation that succinate exacerbates intestinal damage in neonatal NEC models.

We observed that succinate promoted NEC intestinal macrophage polarization to the M1 phenotype. As core members of the innate immune system, macrophages play a critical role in maintaining homeostasis and defending against pathogen infections and their functional status can dynamically change according to microenvironmental signals to form different subtypes, such as proinflammatory M1 type and the anti‐inflammatory M2 type. Specifically, LPS drives macrophage polarization toward the M1 type, whereas interleukin‐4 (IL‐4) induces macrophage polarization toward the M2 type. M1‐type macrophages are able to induce proinflammatory responses and produce the proinflammatory related factors interleukin‐6 (IL‐6), interleukin‐12 (IL‐12), and tumor necrosis factor. In contrast, M2‐type macrophages are able to produce an anti‐inflammatory response and repair damaged tissue [35, 36, 37]. Notably, both clinical and animal experiments have confirmed significant infiltration of macrophages in the NEC‐affected intestine, with an abnormally elevated proportion of the M1 subtype [15, 38, 39]. Multiple studies have shown that succinate can regulate the polarization direction of macrophages. For instance, in a mouse model of Crohn's disease, succinate significantly upregulated the mRNA levels of the M1‐type markers *Cd11c* and *iNOS* [34]; in a mouse model of acute lung injury induced by intestinal ischemia‐reperfusion, succinate also induced alveolar macrophage polarization toward the M1 type as indicated by an increase in M1‐type related markers and a decrease in M2‐type markers [26]. Our results further validated the role of succinate in the regulation of macrophage polarization. In the succinate‐treated group, the protein expression of the M1 marker iNOS was upregulated, whereas the M2 marker ARG1 was downregulated compared to untreated NEC mice. Furthermore, the mRNA expression levels of both *iNOS* and *Arg1* were consistent with their respective protein levels. Immunofluorescence showed that succinate intervention significantly increased the proportion of intestinal F4/80^+^/CD86^+^ (M1‐type) coexpression in NEC mice, whereas the opposite trend was observed for F4/80^+^/CD206^+^ (M2‐type) expression. The expression levels of CD86 and CD206 proteins determined by ELISA were consistent with the coexpression trends of F4/80^+^CD86^+^ and F4/80^+^CD206^+^ determined by immunofluorescence. In conclusion, our results suggest that succinate promotes the polarization of NEC neonatal mouse intestinal macrophages toward the M1 type, as a result that is consistent with existing studies in which succinate regulates the direction of macrophage polarization.

We further found that SUCNR1 is essential for succinate‐promoted polarization of NEC intestinal macrophages. SUCNR1 is a natural and unique receptor for succinate [40, 41] and is expressed on macrophage membranes in various tissues, such as the intestine, acute lung injury, and lung tumors [26, 34, 42]. Studies have shown that the upregulation and activation of SUCNR1 in intestinal macrophages increases the expression of proinflammatory cytokines and M1 macrophage marker genes, whereas a lack of *SUCNR1* attenuates their expression, thereby protecting mice from colitis [34, 43]. Similarly, our results revealed that exogenous succinate treatment enhanced SUCNR1 expression in intestinal macrophages from the NEC model and the RAW264.7 cell line, and that the receptor‐specific neutralizing antibody effectively blocked the succinate‐induced M1 polarization phenotype. However, the biological effects of SUCNR1 may be tissue‐specific, such as, in the renal interstitial fibrosis model, where the receptor exacerbates pathological injury by activating profibrotic M2‐type macrophages [44], and in the lung cancer microenvironment, where SUCNR1‐mediated M2 polarization of tumor‐associated macrophages promotes malignant metastasis [42]. This functional plasticity suggests that the macrophage response pattern to the SUCNR1 signaling pathway may be regulated by local microenvironmental factors and the underlying mechanisms require further elucidation.

Our study also revealed that the PI3K/AKT signaling pathway may mediate succinate‐promoted macrophage polarization. Succinate activates intracellular signaling by binding to SUCNR1, thereby exerting its biological functions [34, 45]. The PI3K/AKT pathway is a classical prosurvival pathway in all types of cells and it contains many downstream components, such as the FOX family, glycogen synthase kinase 3 (GSK3), and hypoxia‐inducible factor‐1a (HIF1a) [46, 47, 48]. This pathway also plays an important role in macrophage polarization, with studies showing that an increase in succinate can trigger tumor‐associated macrophage polarization through the PI3K/AKT pathway in lung cancer [42]. Another study has showed that succinate can influence the polarization of alveolar macrophages through this pathway in lung injury after intestinal ischemia‐reperfusion [26]. Our study revealed that succinate treatment of NEC pups and LPS‐induced RAW264.7 cells activated the PI3K/AKT pathway and affected macrophage polarization; in contrast, inhibition of the pathway with LY294002 reversed these effects. These findings suggest that succinate may mediate the effects of M1‐type macrophage polarization through this signaling pathway, which in turn exacerbates the pathological process of NEC. This phenomenon is consistent with previous findings on the regulation of macrophage polarization by succinate through the PI3K/AKT signaling pathway.

This study has several limitations. Firstly, numerous studies have shown that SUCNR1 is the natural and unique receptor for succinate [40, 41] and that succinate exerts its effects by binding to this receptor. In this study, we used neutralizing antibodies against SUCNR1 and found that they could partially eliminate the effects of SUCNR1. However, it should be emphasized that, compared with the neutralizing antibody blockade strategy, *SUCNR1* knockout can better reveal the regulatory mechanism of this receptor in NEC. The team plans to construct *SUCNR1* knockout mice to further elucidate the key role of *SUCNR1* in the pathogenesis and pathological process of NEC. In addition, we verified the effective inhibition of pathway activation by using PI3K/AKT pathway inhibitors. However, considering the possible nonspecific effects of LY294002, we intend to use a more specific PI3K inhibitor or construct a *PI3K/AKT* knockout model to further clarify its mechanism of action. Furthermore, the present study lacked data on the correlation between the SUCNR1 receptor and the PI3K/AKT pathway in clinical samples, which will be supplemented in future studies.

**FIGURE 9:**
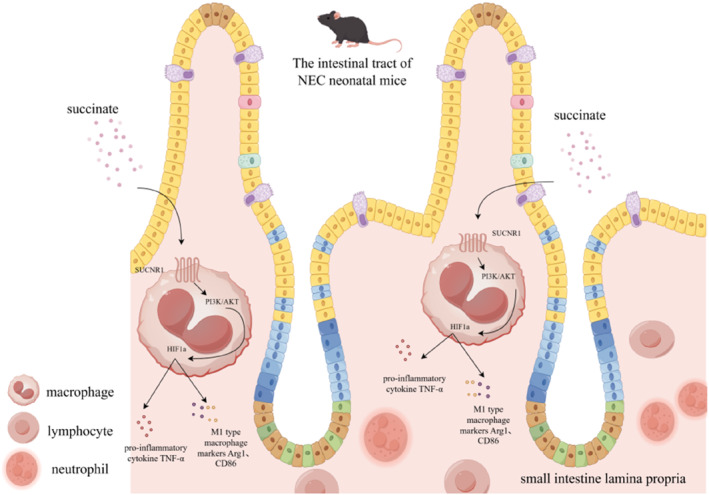
Mechanistic diagram of the mechanism by which succinate promotes intestinal macrophage polarization in NEC neonatal mice.

In summary, our findings indicate that succinate may activate the PI3K/AKT signaling pathway through SUCNR1 to promote M1 polarization of NEC intestinal macrophages. The activation of this pathway can aggravate the progression of NEC. Our study provides a new perspective for understanding the pathogenesis of NEC and offers a possible target for further treatment of NEC in the future. The mechanism by which succinate promotes the polarization of intestinal macrophages in NEC neonatal mice is shown in Figure 9.

## Author Contributions

All the authors made substantial contributions to the study. Lei Bao and Lu‐Quan Li conceived and designed the study, supervised the project, reviewed and edited the article. Sha Liu and Fang‐Ling Tang performed the research and analyzed the data. Sha Liu wrote the original draft. Xiao‐Lin Yan, Xiao‐Chen Liu and Qing Ai contributed new methods or models and supported the experimental progress.

## Ethics Statement

The Animal Ethics Committee of Chongqing Medical University approved all the experiments performed in this study (No. CHCMU‐IACUC20240111013).

## Conflicts of Interest

The authors declare no conflicts of interest.

## Data Availability

The authors have nothing to report.
